# Retinopathy of prematurity: incidence report of outliers based on international screening guidelines

**DOI:** 10.1186/s40942-019-0203-x

**Published:** 2019-12-12

**Authors:** Juan Carlos Romo-Aguas, Ana González-H.León, Miroslava Paolah Meraz-Gutiérrez, María A. Martínez-Castellanos

**Affiliations:** 1 Retina Department, Asociación para Evitar la Ceguera en México I.A.P, Mexico City, Mexico; 2 Retina Service, Asociación para Evitar la Ceguera Hospital ‘‘Luis Sánchez Bulnes’’ I.A.P., Vicente Garcia Torres No. 46 Coyoacán, 04030 Mexico, D.F. Mexico

**Keywords:** Retinopathy of prematurity, Epidemiology blindness, Neonatal intensive care unit

## Abstract

**Aim:**

The objective of this study is to report the incidence of retinopathy of prematurity (ROP) outliers that fall outside the screening guidelines of the American Academy of Ophthalmology (AAO) in our country.

**Methods:**

A retrospective review of 503 records of newborns evaluated in our institution between January 2011 and March 2017. We analyzed the data by subgroups based on gestational age (GA), birth weight (BW) and stage, focusing on the outliers that don’t meet the criteria of the screening AAO guidelines (GA ≤ 30 weeks, BW ≤ 1500 g).

**Results:**

Of the 503 records, 352 had some degree of ROP, 91.76% being bilateral, and 26.2% require treatment. The mean GA at delivery was 30.56 ± 2.33 weeks, and the mean BW was 1287.90 ± 338.52 g. For the current AAO/AAP ROP screening, 19.9% were outliers, of which (57%) had ROP diagnosis and (38%) required treatment.

**Conclusions:**

ROP diagnosis in newborns of BW > 1500 g or GA > 30 weeks is not uncommon in Mexico, and it is important to take this into account to adjust the selection criteria on each population to reach all the infants at risk.

## Background

Retinopathy of prematurity (ROP) is a potentially blinding condition characterized by abnormal vascular growth of the immature retina that affects preterm infants [1]. Each year about 15 million babies are born prematurely [2]. Developing countries are now seeing a spike due to the higher premature birth rates, decreased access to neonatal resources, and possibly due to lack of awareness or training of healthcare professionals; in Latin America is the leading avoidable cause of childhood blindness [3].

The latest ROP screening guidelines published by the American Academy of Pediatrics and the American Academy of Ophthalmology (AAO/AAP), recommend that all infants with gestational age (GA) of ≤ 30 weeks and/or ≤ 1500 g of birth weight (BW), or with unstable clinical course should be screened [4].

In our country, the Mexican Secretary of Health (SSA-Spanish acronym) made an update in ROP screening guidelines in July 2015, increasing the threshold in order to screen all newborns that were born ≤ 34 weeks at BW or/and ≤ 1750 g of BW [5]. The study of Flores-Santos in 2007 and 2 report prevalence series in Mexico are the basis of this adjustment [6–8], in which they found patients that had ROP and require treatment that fell outside the AAO/AAP guidelines.

The purpose of this study is to report the incidence of ROP in infants who do not fall within the AAP/AAO screening criteria in our center, to help identify infants at risk for ROP in Mexico and other middle-income countries.

## Methods

A retrospective review of all the records of ROP screening program in our institution, which englobe 503 infants who underwent evaluation at the Asociación para Evitar la Ceguera en México, “Dr. Luis Sánchez Bulnes” I.A.P. between January 2011 and March 2017. The patients are referred to us from inside Mexico City, and other regions of the country (southeast and central more frequently); and this is because some of the states don´t have a proper screening program (personel or training) that could reach all pre-term infants.

A considerable percentage of these patients arrive at end-stages of the disease because based on the newborns health they are brought on incubators, only if the are clinically stable to be transported on an ambulance; or sometimes after they are discharged, and by that time it could be already too late for timely diagnosis and treatment. It is important to know that not all patients who arrive at the hospital are referred with a diagnosis of ROP, but are sent due to risk factors.

At our hospital triage, every infant with a history of pre-term birth, low birth weight or any perinatal complications is sent to the pediatric retina department, where a trained retina specialist team do a complete ophthalmologic examination (this evaluation is performed by two pediatric retina specialist MAMC or LCE and retina fellows tutored by them); including all the past medical history (gestational age, birth weight at delivery, demographic information, medical history, previous interventions, other systemic associations, and prior treatment).

After this evaluation is done, we use a registration format (see Additional file 1) where we include all relevant medical history and the ophthalmologic findings including ROP status; according to the International Classification of ROP (ICROP) [9], with the maximum grade of retinopathy defined as the highest stage and lowest zone on each eye.

**Fig. 1:**
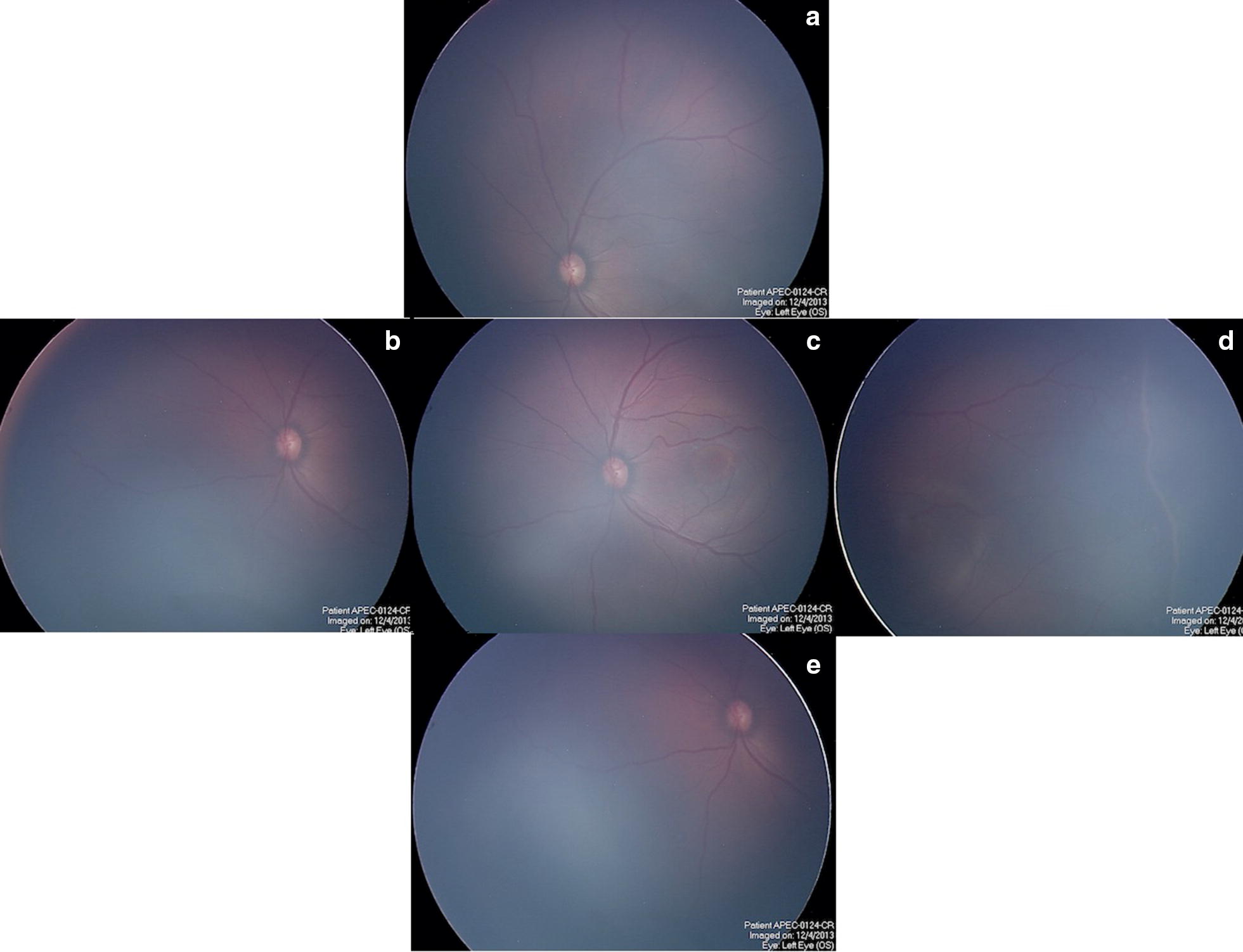
Example of the protocol pictures taken at every visit

Also as part of our follow-up procedures, we take 5 protocol pictures (one centered on the optic nerve, superior, inferior, nasal and temporal) every visit to compare one and other, with The *RetCam II* (*Clarity* Medical Systems, Pleasanton, Calif) (see Fig. 1) and recently we also use ultra wide field fundus camera Optos Daytona ^®^ (Optos, Dunfermline, United Kingdom).

When non-surgical treatment is needed, we use antiangiogenic therapy with either intravitreal injection of Bevacizumab or Ranibizumab. Our threshold for antiangiogenic treatment is based on the AAP/AAO 2018 recommendations [4], Zone I, any stage with plus disease; Zone I Stage 3 without plus disease; Zone II Stage 2 or 3 with plus disease. We also include Zone II Stage 3 without plus disease.

When no treatment is required, the infant is monitored closely until complete vascularization of the retina is achieved. For cases of ROP that do not regress or are too advanced for antiangiogenic treatment, laser therapy or vitrectomy is performed. Indications for treatment followed the guidelines from The Early Treatment for Retinopathy Of Prematurity (ETROP) study [10].

Patients were grouped according to GA, BW, and stage of ROP for analysis. The study mainly focused on infants that were outliers to the AAO/AAP screening guidelines (GA ≤ 30 weeks and BW ≤ 1500 grams) in which a diagnosis of ROP was made.

## Results

We report all the ROP screening records from our hospital from January 2011 to March 2017, with a total of 503 infants. The distribution was very similar in both genders, being 259 (51.5%) females and 244 (48.5%) males. The mean GA at delivery was 30.56 ± 2.33 weeks (23–37 weeks) and the mean BW was 1287.90 ± 338.52 g (540 and 2932 g).

**Fig. 2:**
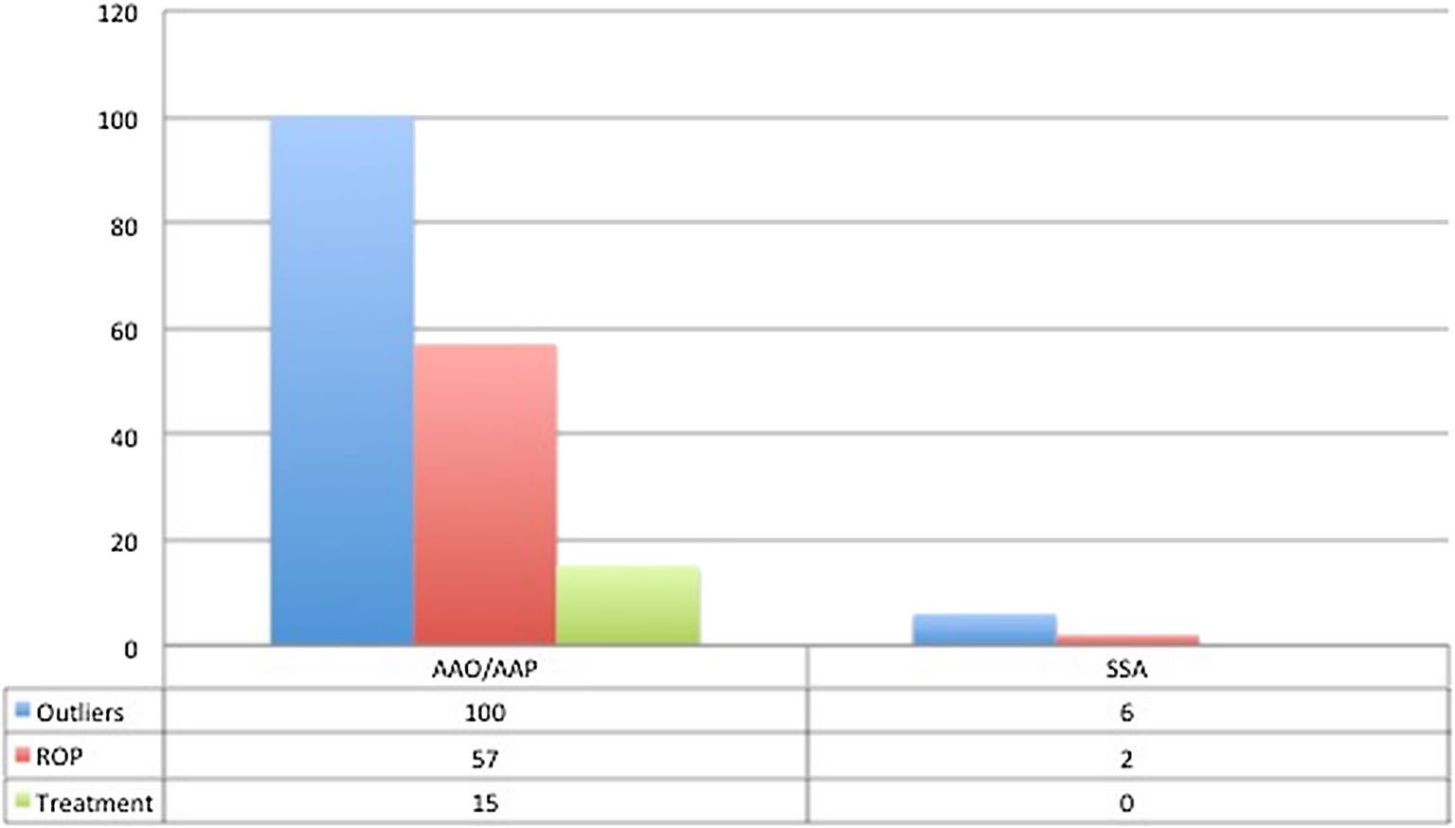
Graph displaying the number of outliers based on the different ROP screening guidelines

Throughout this period, 70% of the screenings were detected with some degree of ROP on either eye, which represents 352 of 503 newborns; 91.76% being bilateral (323/352). In Tables 1 and 2, we present the distribution of this population, dividing them into groups based on BW and GA at birth (see Fig. 2).

**Table 1:** Demographic data presenting gestational age in relation to ROP diagnosis

Stage	Gestational age (weeks)					
	≤ 30		30.1–33.6		≥ 34	
ROP diagnosis:	219		131		24	
	Treated	Non-treated	Treated	Non-treated	Treated	Non-treated
1	5	43	2	35	0	9
2	12	62	3	35	0	5
3	53	0	20	1	3	0
4	18	5	4	9	3	2
5	5	12	11	2	0	2
Total	93	122	49	82	6	18

**Table 2:** Demographic data presenting birth weight in relation to ROP diagnosis

Stage	Birth weight (g)					
	≤ 1500		1501–1999		≥ 2000	
ROP diagnosis:	306		60		8	
	Treated	Non-treated	Treated	Non-treated	Treated	Non-treated
1	8	66	0	21	0	2
2	14	92	1	11	0	1
3	64	0	11	1	2	0
4	20	15	5	6	0	1
5	7	20	0	4	0	2
Total	113	193	17	43	2	6

The distribution based on the ROP stage at the time of evaluation was: 94 infants were on stage 1 (18.7%), 117 on stage 2 (23.3%), 77 on stage 3 (15.3%), 44 on stage 4 (8.8%), 32 on stage 5 (6.4%); and 132 infants (26.2%) required and received treatment.

For our data analysis, we realized Kolmogorov–Smirnov test to determine if the distribution of the variables GA and BW was normal, which both result < 0.000. Also, we calculate the area under de curve for these two variables for GA was 0.337, and for BW 0.369.

**Fig. 3:**
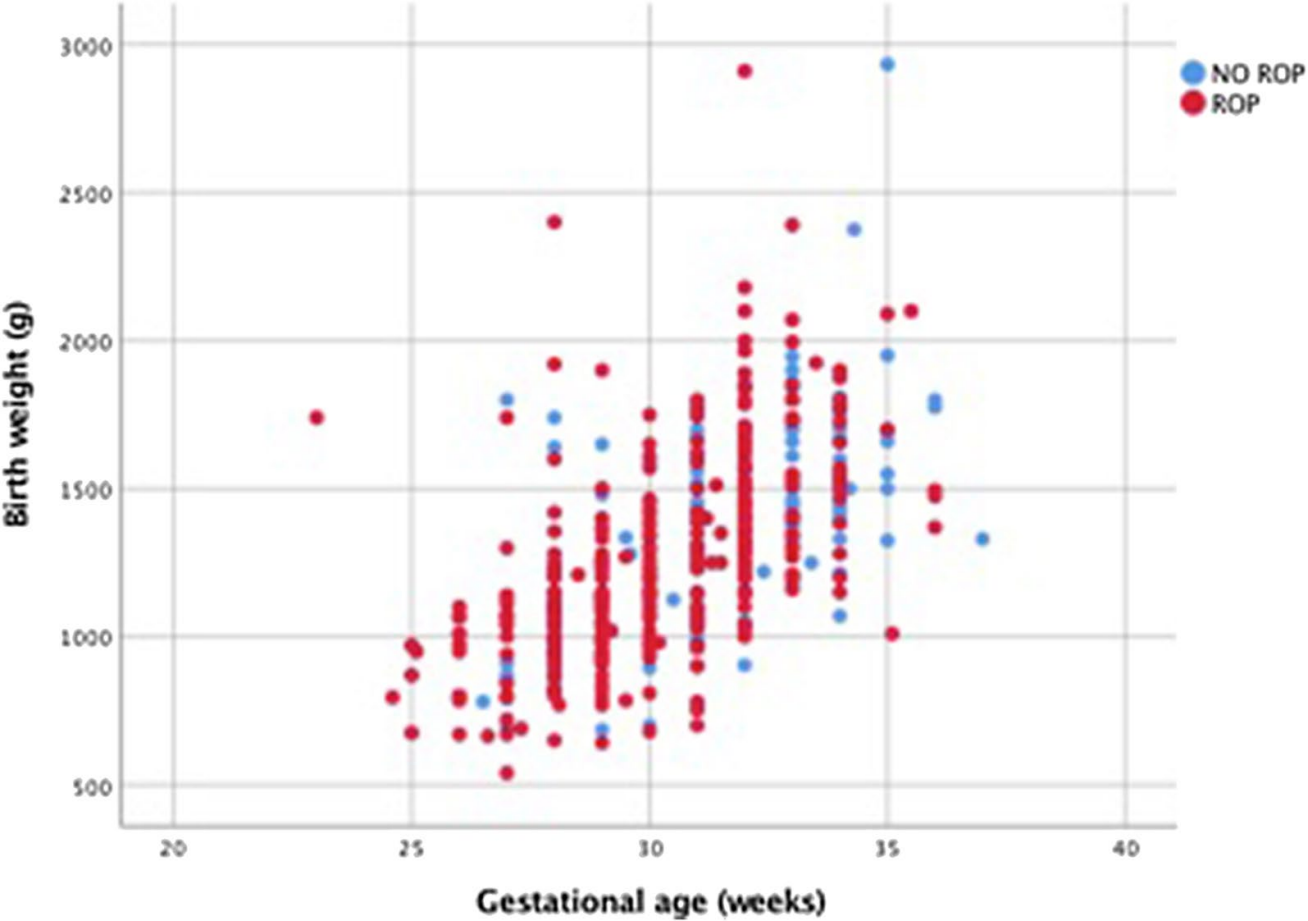
Birth weight and gestational age distribution based on ROP diagnosis

In order to find outliers, we group the populations that meet each of the guidelines. For the current AAO/AAP ROP screening, 100 infants (19.9%) would be considered outliers, of which 57 (57%) had ROP diagnosis and 15 (38%) required treatment. And for the SSA guidelines employed in our country, only 6 patients (1.2%) would be left out as outliers; and only 2 (33.3%) had ROP diagnosis and non of them require treatment (see Fig. 3).

## Discussion

We reviewed 503 charts of infants screened for ROP diagnosis from January 2011 to March 2017. The main purpose of the study was to determine how many infants would be left out if we would base our screening program only in AAO/AAP guidelines [4]. The source to determine the threshold of these guidelines comes from industrialized countries but, previews series in Mexico from 2006 to 2008 [6, 7, 11] report ROP patients in newborns > 32 weeks and GA of 2000 g. For this reason the SSA in 2015 increase the threshold to 34 weeks of GA and 1750 g BW.

The screening criteria used in developed nations may no be applicable for middle-income countries. For example, Gilbert et al. show that if the United Kingdom 2008 screening criteria were applied, an overall of 13% of newborns in middles- and low-income countries would not be examined (< 32 weeks or < 1500 g) [12]. Zimmermann et al. presented the prevalence of ROP in Latin America throughout 2000–2010, ranging from 6.6 to 82% at any stage and of severe ROP from 1.2 to 25% based on AAO/AAP guidelines [13]. Also in Freitas et al., recently reported that in a series of 10 years only reported 8/602 patients with > 32 weeks or > 1500 g [14]. In other middle-income countries, like India, a report showed that the incidence of infants > 1500 g treated for threshold ROP with cryotherapy was 15.3% [15]. And in Lithuania, 54% of the infants needing treatment for ROP were > 1500 g at birth [16].

We found that 19.9% of the screened newborns fell out of the AAO/AAP 2018 guidelines, 57% of these outliers had some degree of ROP, and 38% required treatment. Compared to the SSA guidelines were only 1.2% are outliers, and only 2 (33%) patients had ROP. None of them required treatment. One of the factors associated could be that in neonatal care units with lower survival rates in middle or low-income countries, our selection criteria for screening should be expanded [17].

Whatever the reason for this variability, the difference of outliers between the two guidelines is striking, and it is important to take this into account to adjust the selection criteria on each population. Within the limitations of the study, we have the retrospective nature and the selection bias. As a tertiary health care center, the majority of our patients referred for ROP screening require treatment, probably because stage 1 or 2 are followed up in second-level hospitals.

## Conclusions

In conclusion, ROP diagnosis in newborns of BW > 1500 g or GA > 30 weeks is not uncommon in Mexico and other developing countries. Based on our population, applying the SSA guidelines established in our country in 2015 only 1.2% of the patients would be left out, compared to 57% with AAO/AAP guidelines. And modification of the current screening guidelines in other middle-income countries could be useful to include infants at risk.

## Supplementary information

**Additional file 1.** The format presented as additional material was made by the personnel at our hospital in the admission and follow-ups of every infant that undergoes ROP screening. It contains the following sections: patient ID number, BW, GA, risk factors, a diagram based on ICROP classification, and finally diagnosis/treatment section.

## Data Availability

The database used during the current study is available from the corresponding author on reasonable request.

## Abbreviations

ROP: retinopathy of prematurity
AAO/AAP: American Academy of Pediatrics and the American Academy of Ophthalmology
GA: gestational age
BW: birth weight
SSA: Mexican Health Secretary (Spanish acronym)
ICROP: International Classification of Retinopathy of Prematurity
ETROP: the early treatment for retinopathy of prematurity
