# Supplementary figures and images for: Retinopathy of prematurity: incidence report of outliers based on international screening guidelines
Source: Int J Retina Vitreous. 2019 Dec 12;5(Suppl 1):53. doi: 10.1186/s40942-019-0203-x (PMC6907106; doi:10.1186/s40942-019-0203-x)

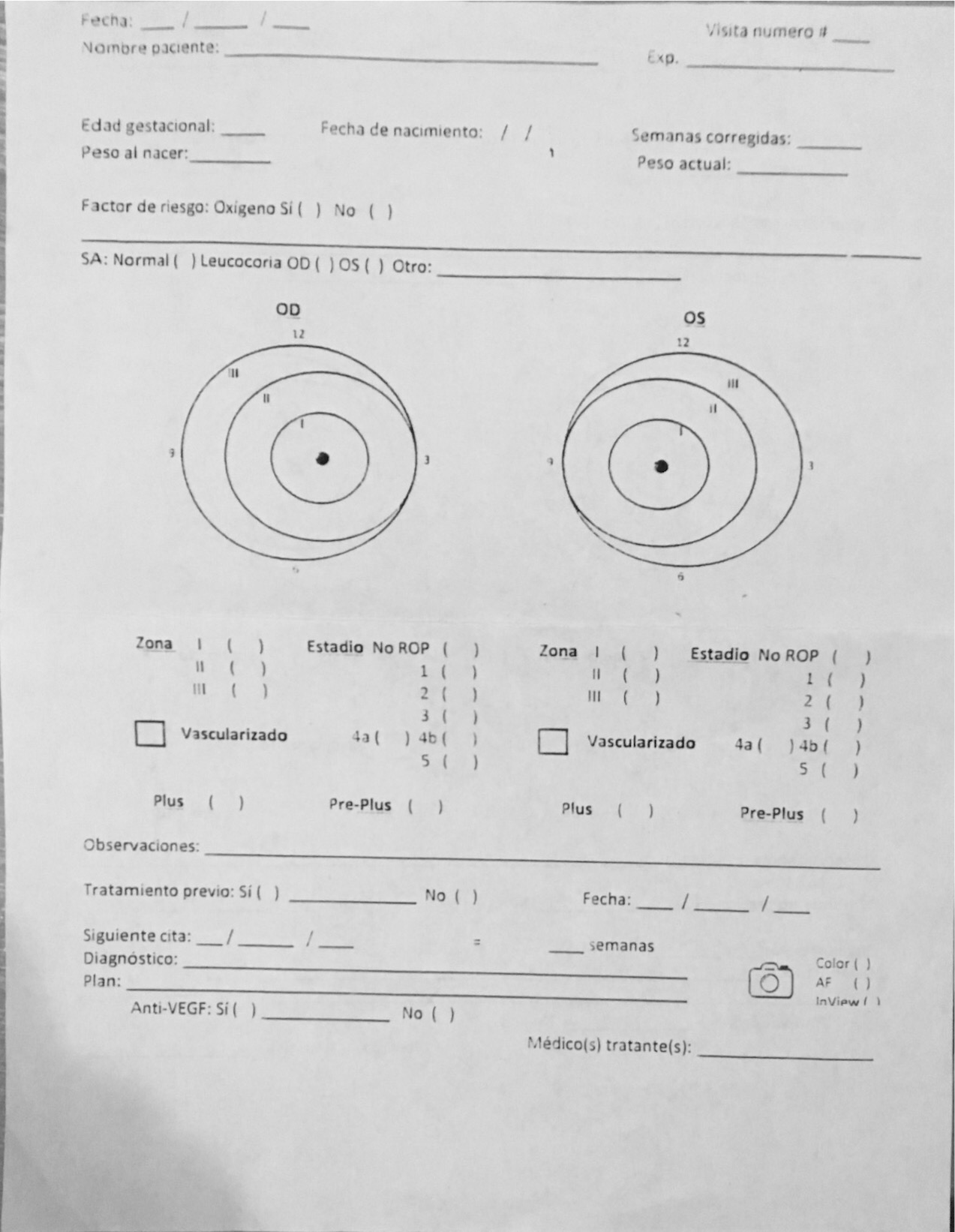

Supplement: Supplementary file 1 — Additional file 1. The format presented as additional material was made by the personnel at our hospital in the admission and follow-ups of every infant that undergoes ROP screening. It contains the following sections: patient ID number, BW, GA, risk factors, a diagram based on ICROP classification, and finally diagnosis/treatment section. [file 40942_2019_203_MOESM1_ESM.jpg]
